# Supplementation of *in vitro* culture medium with FSH to grow follicles and mature oocytes can be replaced by extracts of *Justicia insularis*

**DOI:** 10.1371/journal.pone.0208760

**Published:** 2018-12-07

**Authors:** Gildas Tetaping Mbemya, Jesus Cadenas, Naiza Arcângela Ribeiro de Sá, Denise Damasceno Guerreiro, Nathalie Jiatsa Donfack, Luis Alberto Vieira, Francisca Geovania Canafístula de Sousa, Benner Geraldo Alves, Carlos Henrique Lobo, Francielli Weber Santos, Francisco das Chagas Lima Pinto, Otília Deusdênia Loiola Pessoa, Johan Smitz, Pierre Comizzoli, José Ricardo Figueiredo, Ana Paula Ribeiro Rodrigues

**Affiliations:** 1 Laboratory of Manipulation of Oocyte and Ovarian Preantral Follicles (LAMOFOPA), Faculty of Veterinary (FAVET), State University of Ceará, Fortaleza, Brazil; 2 Laboratory of Biology of Reproduction, Federal University of Uberlândia, Minas Gerais, Brazil; 3 Laboratory of Animal Physiology, Department of Animal Science, Federal University of Ceará, Fortaleza, CE, Brazil; 4 Laboratory of Reproduction Biotechnology (Biotech), State of University of Pampa, Uruguaiana, Brazil; 5 Laboratory of Phytochemical Analysis of Medicinal Plants (LAFIPLAN), Federal University of Ceará, Fortaleza, Brazil; 6 Follicle Biology Laboratory, Center for Reproductive Medicine, UZ Brussel, Brussels, Belgium; 7 Center for Species Survival, Smithsonian Conservation Biology Institute, Front Royal, VA, United States of America; Faculty of Animal Sciences and Food Engineering, University of São Paulo, BRAZIL

## Abstract

The present study evaluated the effect of supplementing *in vitro* culture medium with *J*. *insularis* compared to FSH on isolated secondary follicles and *in vitro* maturation of oocytes from those follicles. Secondary follicles were isolated from sheep ovaries and individually cultured for 18 days in α-MEM^+^ (*Control*), α-MEM^+^ supplemented with 100 ng/mL recombinant bovine follicle stimulating hormone (FSH) or with 0.3, 1.25, or 2.5 mg/mL of *J*. *insularis* extract (JI0.3, JI1.25, and JI2.5, respectively). Culture medium collected every 2 days was used to measure ROS levels. At the end of the culture period, cumulus oocytes complex (COCs) were collected and matured *in vitro*. Follicular walls were used for mRNA quantitation. JI0.3 led to a higher (P < 0.05) percentages of intact follicles than other groups after 18 days of culture. While follicular diameter remained unchanged from Day 6 onwards with JI0.3 and FSH, percentages of antral cavity formation were higher (P < 0.05) with JI0.3 at Day 6 than in all other treatments. No differences were observed between controls and treatment groups regarding ROS levels and mRNA expression of genes. Viability of resulting oocytes was higher (P < 0.05) in JI0.3 compared to FSH. Interestingly, in control experiment, supplementation of maturation medium with JI0.3 led to higher (P < 0.05) percentages of metaphase II compared to controls. Although more validations will be needed, it seems that this natural extract could be used as a cheap and easily available alternative to commercial FSH.

## Introduction

The secondary follicles, the last category of preantral follicles (PF), according to Araújo et al. [1], is an excellent source of potentially fertilizable oocytes; however, the *in vitro* development of these follicles has been a great challenge to produce fully grown and competent oocytes. To date, live birth after the *in vitro* culture of PF has been reported only in mice [2–4]. In large mammals, on the other hand, results are still modest and may be summarized in the production of a variable number of embryos in buffaloes [5], sheep [6] and goats [7, 8].

Several variables may affect the outcome of *in vitro* follicle culture such as the culture base media composition [9, 10] and supplementation [11]; the animal model [8]; culture system [1, 12]; follicular category [13, 14] and reactive oxygen species (ROS) production [15], leading to oxidative stress. Many authors have shown that an increase of glutathione peroxidase (GPx) can represent a cellular transcriptional response to ROS [16], such as an activation or silencing of genes encoding antioxidant defense enzymes, growth and progression of meiosis [17]. To date it still is a challenge to reach a good balance between all components of the culture medium, getting the right concentrations and interaction that should exist between the components of the medium.

The addition of supplements (energy substrates, antioxidants, hormones and/or growth factors) to alpha modified minimal essential medium (α-MEM) is necessary to enable acceptable rates of follicular growth, oocyte viability and maturation [18] in caprine [19, 20] and ovine species [9, 21]. In an attempt to improve secondary follicles development, oocyte viability and maturation, natural supplements may be added to the culture media such as *Amburana cearensis* [22, 23], anethole [19] and rutin [24]. Although these studies have not involved investigation related to gene expression, it is known that *GPx* [25], *kit ligand* (KL—[13]), *cyclin B1* (CCNB1 –[26]) and *hyaluronan synthase 2* (HAS2 –[27] genes are expressed in mature oocytes. Therefore, the identification of these genes in oocytes may be a good indicator of an *in vitro* culture system's viability for secondary follicles.

*Justicia insularis* T. Anders (Acanthaceae) is an herbaceous and perennial plant of 30–75 cm high with opposite ascending branches, widely distributed in the tropical area of Africa [28]. Besides alkaloids, glycosides, polyphenols and triterpenoids, studies undertaken by Telefo et al. [29] and Goka et al. [28] revealed the presence of flavonoids in their leaves which acts as natural antioxidant and FSH-like compound. In fact, a literature survey showed that several studies performed with *Justicia* species have revealed the presence of the aforementioned secondary metabolites. It is worth mentioning that major part of these studies was done by phytochemical screening. In the Western region of Cameroon, *J*. *insularis* is used in association with the leaves of three others medicinal plants (*Aloe buettneri*, *Hibiscus macranthus* and *Dicliptera verticillata*), to treat dysmenorrhoea and some cases of women infertility. This aqueous extract mixture has also been proven, in a series of studies to induce ovarian steroidogenesis and folliculogenesis in female rats [29–31]. In a recent study performed by our team, the aqueous extract of *J*. *insularis* has successfully promoted beneficial effects on morphology, activation and growth of PF (primordial, intermediary, primary and secondary) enclosed in ovarian tissue cultured *in vitro* for 7 days [32]. To date, due to their widely distribution and cost compared to chemical product, there is an increase interest in natural products that prevent oxidative damages, promote follicular growth and oocyte maturation.

Considering the positive results of *J*. *insularis* on the reproductive function *in vivo* [33] and *in vitro* culture of ovarian tissue [32], previously reported, in a general way, the aim of this study was to investigate the effect of different concentrations of the aqueous extract of *J*. *insularis* on the behavior of secondary follicles after 18 days of *in vitro* culture. In addition, we evaluate the effect of the extract on *in vitro* maturation of oocytes from *in vivo* grown follicles. Therefore, were analyzed: *a)* follicular morphology; *b)* ability to grow and form the antral cavity; *c)* gene expression (GPx, KL, CCNB1 and HAS2) in the follicular wall; *d)* ROS levels in the follicles culture medium and finally, *e)* the viability and *in vitro* maturation of oocytes from *in vitro* and in *vivo* grown secondary follicles.

## Materials and methods

This study was approved and performed according to the recommendations of the Committee of Animal Handling and Ethical Regulation from the State University of Ceará (N° 6004720/2015).

### Chemicals and media

Unless mentioned otherwise, the culture media and other chemicals used in the present study were purchased from Sigma Chemical Co. (St. Louis, Mo, USA).

### Plants materials and phytochemical investigation from leaves of *J*. *insularis*

The fresh leaves of *J*. *insularis* previously identified in the National Herbarium of Cameroon under voucher specimen code 34997 [29] were collected in Western Cameroon (Batoufam subdivision, Upper-Plateau division, 5° 21′ Nord 10° 24′ East, Altitude 1515 m). The fresh leaves were then dried at room temperature in the shade. Subsequently, the plant extract decoction was prepared according to the protocol described by Telefo et al. [33]; briefly, 100 g of powder was submerged in 1.5 L of boiling distilled water for 30 minutes. After cooling, the extract was filtered and dried in a ventilated oven at 45°C. Finally, the plant decoction was lyophilized and kept in the freezer at -20°C. 22.96 g of the lyophilized extract was then diluted in the distilled water to obtain the desired concentrations.

A phytochemical screening of *J*. *insularis*, was performed by thin layer chromatography (TLC), proton nuclear magnetic resonance (^1^H NMR) and liquid chromatography-mass spectrometry (LC-MS), reliable methods used for identification of secondary metabolites.

Firstly, 100 mg of aqueous extract was suspended in MeOH (2 x 10 mL), over sonication for 15 min. The MeOH soluble fraction was evaporation under reduced pressure to yield 1.8 mg of the MeOH fraction (F1), while the insoluble fraction, designed F2, afforded 98.2 mg. 1.0 mg of each fraction (F1 and F2) was solubilized in 1.0 mL of MeOH and H_2_O, respectively, and subsequently applied on silica gel (Merck) chromatography plates (6 x 2 cm) with fluorescence indicator (F254). The samples were applied to the plates with the aid of a capillary tube, and for its development a ternary mixture of n-BuOH/AcOH/H_2_O (6:2:2) was used as the elution system. The plates were immersed in suitable developers and heating to ~100°C, when necessary. Developers specific for each class of secondary metabolites were used: Drangendorffi reagent (A) to identify alkaloids; *α*-naphthol acid solution (B) for glycosylated compounds; cerium sulfate acid/EtOH (C) for flavonoids and terpenoids, and vanillin/EtOH/perchloric solution (D) and EtOH/H_2_SO_4_ solution (E) as universal developers [34, 35]. After this procedure, a sample of the aqueous extract was fractionated on sephadex LH-20 using MeOH/H_2_O 8:2 as solvent of elution to obtain four main fractions; whose ^1^H NMR and LC-MS spectra were obtained.

### Source of ovaries and experimental design

Ovaries from 35 adults (1–3 years old) mixed-breed sheep (*Ovis aries*) were collected at a local abattoir (Guaiúba municipality, Ceará, Brazil) at different times. Immediately postmortem, pairs of ovaries were washed once in 70% alcohol and then twice in minimum essential medium (MEM) plus HEPES (MEM-HEPES). The ovaries were placed into tubes containing 15 mL of MEM-HEPES, supplemented with penicillin (100 μg/mL) and streptomycin (100 μg/mL) and then transported to the laboratory at 4°C within 1 h since they were collected [36]. A total of 342 secondary follicles obtained from those ovaries were randomly *in vitro* cultured for 18 days in five different treatments as described below.

Additionally, to ensure that oocytes respond to *in vitro* maturation (IVM), ovaries from others 40 (1–3 years old) mixed-breed sheep were obtained as previously described and transported at 34°C during 1–2 h. We performed three replicates to obtain a total of 328 complexes oocyte cumulus cells (COCs) from antral follicles which were randomly distributed into five different IVM protocols as described below.

### Isolation, selection and *in vitro* culture of sheep secondary follicles

In the laboratory, ovarian cortical slices (1–2 mm thick) were cut using a surgical blade and placed in MEM-HEPES. Then, secondary follicles (approximately 150–250 μm in diameter) were visualized under a stereomicroscope (SMZ 645 Nikon, Tokyo, Japan) and manually dissected from the slices of ovarian cortex using 26-gauge needles. After isolation, only secondary follicles with a visible oocyte, surrounded by granulosa cells, an intact basement membrane and no antral cavity [36] were selected to *in vitro* culture in different media conditions, corresponding to five different treatments. Therefore, secondary follicles were cultured in α-MEM (M5650, pH 7.2–7.4), supplemented with 3 mg/mL of bovine serum albumin (BSA), ITS (10 μg/mL insulin, 5.5 μg/mL transferrin, 5 ng/mL selenium), 2 mM of glutamine, 2 mM of hypoxanthine, 50 ng/mL of Leukemia Inhibitory Factor (LIF) and 50 ng/mL of Kit Ligant (KL), referred as α-MEM^+^ [37], considered the *Control*. Others follicles were *in vitro* cultured in α-MEM^+^ supplemented with 100 ng/mL recombinant bovine follicle stimulating hormone (FSH) or 0.3, 1.25, or 2.5 mg/mL of *J*. *insularis* (JI0.3, JI1.25, and JI2.5, respectively). The concentration of recombinant bovine FSH and *J*. *insularis* were chosen based on previous studies performed in our laboratory [8, 32]. The secondary follicles were individually cultured in 100 μL drops of five different (α-MEM^+^, FSH, JI0.3, JI1.25, and JI2.5) culture medium on Petri dishes (60 ×15 mm; Corning, USA) under mineral oil for 18 days at 39°C in 5% CO_2_ in air. Fresh medium was prepared immediately before use and incubated for 2 h prior to use, with 60 μL medium being replaced in each drop every 2 days [24].

After *in vitro* culture, follicular development and ROS levels were analyzed. In addition, oocytes recovery from follicles at the end of the culture period were *in vitro* matured for evaluation of oocyte viability, meiotic stages, meiotic resumption, and genes expression on follicular walls.

#### Morphological and follicular development (diameter and antrum formation) evaluation

Follicles were classified according to their morphology as *intact* (no rupture of basement membrane), *extruded* (follicles were those that underwent rupture of their basement membrane) or *degenerated* (follicles showed darkened oocyte and/or misshapen granulosa cells). The percentage of morphologically intact follicles and follicular diameter were calculated only in intact follicles. The percentage of extruded follicles was calculated taking into account the total of extruded follicles divided by the total of intact normal follicles. Follicular diameter was calculated as the mean of two perpendicular measures of each follicle every 6 days with the aid of an ocular micrometer attached to a stereomicroscope (SMZ 645 Nikon, Tokyo, Japan; 100 x magnification). The average follicular daily growth was calculated as follows: the diameter of morphologically intact follicles on the last day they were intact minus their diameter at day zero divided by the number of days they remained intact. Antral cavity formation was defined as a visible translucent cavity within the granulosa cell layers [37].

#### Categories of follicular growth velocity

All follicles in each treatment (Control, FSH, JI0.3, JI1.25 and JI2.5) were divided into three categories according to their daily growth as described previously [38]: (1) null growth (–12.7–0.0 μm day –1), follicles that did not grow during the culture, (2) low growth (0.1–12.0 μm day^–^1), follicles that grew up to 12 μm daily and (3) fast growth (12.1–46.7 μm day^–^1), follicles that grew up to 46.7 μm daily.

#### Reactive oxygen species levels

The ROS levels were determined in the conditioned media by a spectrofluorometric method [39], using 2’, 7’ dihydrodichlorofluorescein diacetate (DCHF-DA) assay. Sample aliquot (50 μL) of media collected was incubated with 5 μL of DCHF-DA (1 mM). The oxidation of DCHF-DA to fluorescent dichlorofluorescein was measured for the detection of ROS. The DCF fluorescence intensity emission was recorded at 520 nm (with 480 nm excitation) 2 h after the addition of DCHF-DA to the medium.

#### Quantitative RT-PCR

For evaluation of GPx, KL, CCNB1, HAS2 and GAPDH mRNA expression, total RNA of three pools of 8–10 viable follicular walls (granulosa and theca cells) was extracted using the Trizol reagent method (Invitrogen, Carlsbad, CA, USA) according to the recommendations of the manufacturer and further purified with PureLink RNA Mini Kit (Anbion, Carlsbad, CA, USA). After extraction, RNA concentration was determined using the NanoDrop System (Thermo Scientific NanoDrop Products), performed with 2 μL of material. Before the cDNA synthesis, all samples were standardized with the same amount of RNA to minimize qPCR variability. cDNA synthesis was performed according to the instructions of SuperScript III RT-PCR (Invitrogen, Carlsbad, CA, USA) manual using random primers (Invitrogen, Carlsbad, CA, USA) from 1 ng of total RNA. The gene-specific primers used for the amplification of different transcripts are shown in Table 1. All primers set annealed at 60°C.

**Table 1:** Oligonucleotide primers used for polymerase chain reaction analysis.

Target gene	Primer sequence (5´→ 3´)	Orientation	Genbank accession no.
**GPX**	GCAACCAGTTTGGGCATCAG TAGGGTCGGTCATGAGAGCA	SenseAnti-sense	GI: 004018462 (*Ovis aries*)
**KL**	AGCGAGATGGTGGAACAACTGTCAGTTCTTCCATGCACTCCACAAGGT	Sense Anti-sense	GI: 16580734 (*Capra hircus*)
**CCNB 1**	AGCGGATCCAAACCTTTGTAGTG CAATGAGGATGGCTCTCATGTTTC	SenseAnti-sense	GI: 327679 (*Bos taurus*)
HAS 2	CCTCATCATCCAAAGCCTG ACATTTCCGCAAATAGTCTG	SenseAnti-sense	GI: 174079.2 (*Capra hircus*)
**GAPDH**	ATGCCTCCTGCACCACCA AGTCCCTCCACGATGCCAA	SenseAnti-sense	GI: 298676424 (*Ovis aries*)

GPx: *glutathione peroxidase*; KL: *Kit ligand*; CCNB 1: *Cyclin B1*: HAS 2: *hyaluronan synthase 2*; GAPDH: *glyceraldehyde-3-phosphate dehydrogenase*

The qPCR reaction was performed in quadruplet always using control without cDNA to avoid possible contamination. Evaluations were performed in IQ5 Real-Time PCR Detection System (Bio-Rad, Hercules, CA, USA) using analysis of relative quantification. Detection of PCR products was measured by monitoring the increase in fluorescence emitted by the marker Power SYBR Green PCR Master Mix (Applied Biosystems, Carlsbad, CA, USA). For all amplifications, one dissociation curve (melting curve) was done for the verification of unspecific amplifications arising from contamination was held. The qPCR thermal cycle was as follow: initial denaturation and activation of the polymerase for 15 min at 94°C, followed by 40 cycles of 15 s at 94°C, 30 s at 60°C and 45 s at 72°C. The final extension was for 10 min at 72°C. Quantification of the transcripts of target genes was calculated from the difference of the values of the Ct values (threshold cycle PCR) in relation to transcripts of the endogenous gene, glyceraldehyde 3-phosphate dehydrogenase (GAPDH). First, the mean Ct of each sample, both the target gene and the endogenous gene was determined. From each sample, the subtraction of the mean value of the Ctgene-target to Ctgene-endogene provided the ΔCt. Subsequently, one ΔCt corresponding to a calibrator was chosen, normalizing all values by subtracting the resulting ΔCt chosen, to obtain the ΔΔCt. Finally, the final value of relative quantification was given by 2^-ΔΔCt^, where the calibrator or standard sample chosen was equal to one [40].

### *In vitro* maturation of oocytes from *in vitro* grown secondary follicles

At the end of the culture period (day 18), all morphologically intact and extruded secondary follicles were carefully opened with 26-G needles under a stereomicroscope for oocyte recovery. Only oocytes (≥110 μm) with homogeneous cytoplasm and surrounded by at least one compact layer of cumulus cells were selected for *in vitro* maturation (IVM). The recovery rate was calculated by dividing the number of oocytes (≥110 μm) by the sum of morphologically intact and extruded follicles at day 18 of culture, multiplied by 100. The selected cumulus oocyte complexes (COCs) were washed three times in maturation medium composed of TCM 199 supplemented with 1% BSA, 5 μg/mL LH, 0.5 μg/mL recombinant bovine FSH, 10 ng/mL epidermal growth factor (EGF), 50 ng/mL insulin like growth factor 1 (IGF-1), 1 mM pyruvate, 1 μg/mL estradiol (E2), 100 μM cysteamine and different concentrations of *J*. *insularis* (JI0.3, JI1.25, or JI2.5). After being washed, the COCs were transferred to 100-μL drops of IVM medium (approximately 10 COCs per drop) under mineral oil and then incubated for 40 h at 39°C with 5% CO_2_. At the end of the maturation period, oocytes were stained with 10 μM Hoechst 33342 (483 nm) for the assessment of chromatin configuration.

### *In vitro* maturation of oocyte recovery from antral follicles

To evaluate the effect of *J*. *insularis* on viability and maturation of oocytes grown *in vivo* (from antral follicles), selected COCs which served as a control for IVM of oocytes from secondary follicles grown *in vitro* were recovered from sheep ovary by slicing. The recovered COCs were washed twice with TCM 199 buffered with 25 mM HEPES (TCM 199—HEPES) and antibiotics. Then, only oocytes with homogeneous cytoplasm and surrounded by at least one compact layer of cumulus cells were selected and randomly distributed into five maturation procedures as follows: TCM 199 (*Control*), TCM 199 supplemented with 0.5 μg/mL FSH or with 0.3, 1.25, or 2.5 mg/mL of *J*. *insularis* (JI0.3, JI1.25, and JI2.5, respectively). Furthermore, groups of 20–35 oocytes were cultured in 200–350 μL (10 μL per COCs) maturation medium, for 24 hours in the same conditions mentioned above. At the end of the maturation period, the oocyte viability, meiotic resumption, and meiotic stages were evaluated.

#### Assessment of oocyte viability and chromatin configuration

After IVM, oocyte (grown *in vitro* or *in vivo*) chromatin configuration and viability were assessed by fluorescence microscopy (Nikon, Eclipse 80i, Tokyo, Japan; 400x magnification). Oocytes were mechanically denuded by repeated pipetting and incubated in 100 μl droplets of PBS with 4 μM calcein-AM, 2 μM ethidium homodimer-1 (Molecular Probes Live/dead Viability/Cytotoxicity Kit for mammalian cells L3224, Invitrogen, Karlsruhe, Germany), 10 μM Hoechst 33342, and 0.5% glutaraldehyde. The emitted fluorescent signals of calcein-AM and ethidium homodimer were collected at 488 and 568 nm, respectively. Whereas the first probe detected the intracellular esterase activity of viable cells, the later labeled the nucleic acids of non-viable cells after plasma membrane disruption. Oocyte chromatin was stained by Hoescht 33342 (emission at 483 nm), and classified as germinal vesicle (GV), germinal vesicle breakdown (GVBD), metaphase I (MI), metaphase II (MII), or degenerated (DEG). The oocytes were considered viable when cytoplasm stained with calcein-AM (green) did not show abnormal chromatin configuration and/or did not label with ethidium homodimer (red).

### Statistical analysis

All statistical procedures were carried out with Sigma Plot version 11.0 (Systat Software Inc., USA). Normality and homogeneity of variance were evaluated by Shapiro-Wilk and Levene's tests, respectively. Comparisons of means was performed by Kruskal-Wallis or Wilcoxon-Mann-Whitney tests, when appropriate. The percentage variables were analyzed among treatments and days of culture by chi-square or Fisher’s exact tests. Odds ratio and confidence interval (95%) were calculated to evaluate the effect of follicular growth category on percentages of extrusion. The mRNA expression level was analyzed using the t-Tests. Data were presented as mean (± SEM) or percentage. Statistical significance was defined as P < 0.05 (two-sided).

## Results

### TLC, ^1^H NMR and LC-MS analysis

According to the TLC procedures (S1 Fig), ^1^H NMR and LC-MS data were detected alkaloids, phenol compounds and sugars in the aqueous extract of *J*. *insularis*, corroborating with previous phytochemical reports on the *Justicia* species [34, 35].

### Influence of *J*. *insularis* extract on follicular morphology during *in vitro* culture

**Fig 1:**
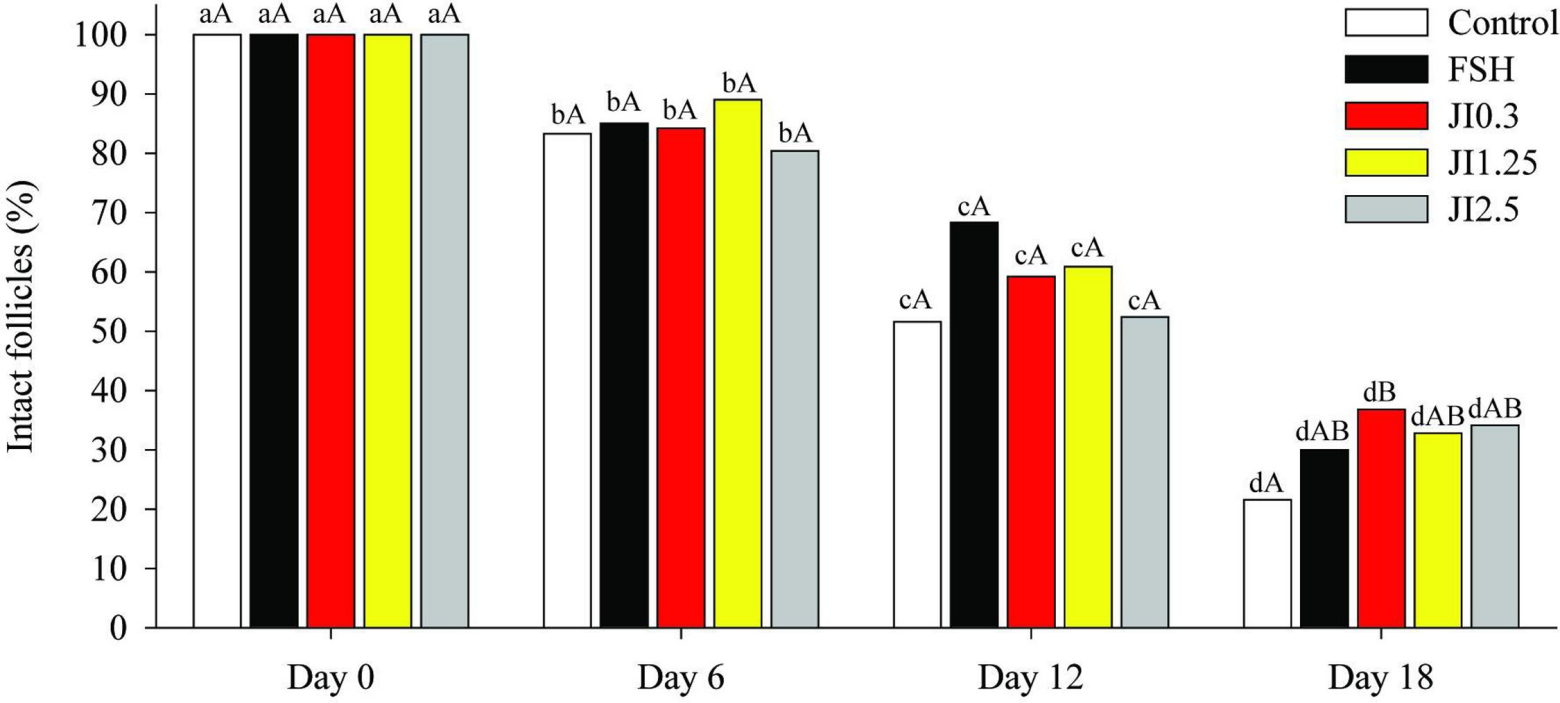
Percentage of morphological intact follicles at different time point (D0, D6, D12, D18) in MEM^+^ (Control), FSH or in *J*. *insularis* 0.3, 1.25 or 2.5 mg/mL (JI0.3, JI1.25 or JI2.5 respectively). D: day, MEM^+^: Minimal essential medium supplemented, FSH: recombinant bovine follicle stimulating hormone, JI: *J*. *insularis*. A total of 342 secondary follicles were distributed in the different treatments (n = 6 replicates). ^a,b,c,d^ Different letters denote significant differences among time for a given treatment group (P < 0.05). ^A,B^ Different letters denote significant differences among treatment groups within the same time point (P < 0.05).

Over the 18 days of the culture period, a significant decrease (P < 0.05) in the percentage of intact follicles was observed in all treatments. However, JI0.3 led to a higher (P < 0.05) percentage (36.8%) of intact follicles than in control treatment (21.6%) at the end of the culture period (Fig 1).

**Fig 2:**
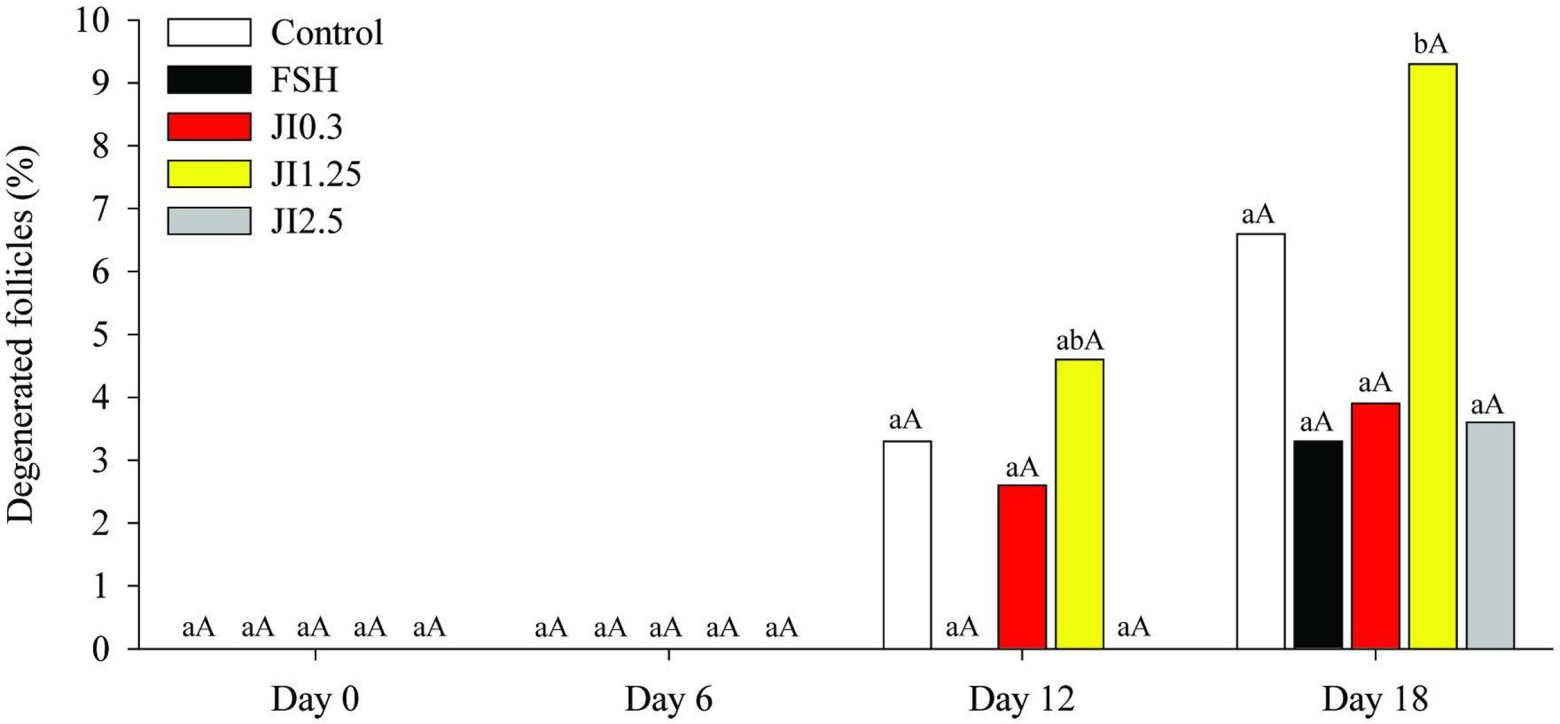
Percentage of degenerated follicles at different time point (D0, D6, D12, D18) in MEM^+^ (Control), FSH or in *J*. *insularis* 0.3, 1.25 or 2.5 mg/mL (JI0.3, JI1.25 or JI2.5 respectively). D: day, MEM^+^: Minimal essential medium supplemented, FSH: recombinant bovine follicle stimulating hormone, JI: *J*. *insularis*. A total of 342 secondary follicles were distributed in the different treatments (n = 6 replicates). ^a,b,c,d^ Different letters denote significant differences among time for a given treatment group (*P* < 0.05). ^A,B^ Different letters denote significant differences among treatment groups within the same time point (P < 0.05).

No degenerated follicles were observed at D0 and D6. Small proportions of follicles were degenerated at D12 across all treatments. At D18, only JI1.25 treatment significantly increased the percentage of degenerated follicles compared to D0 (Fig 2).

### Influence of *J*. *insularis* extract on follicular growth and antral formation during *in vitro* culture

**Fig 3:**
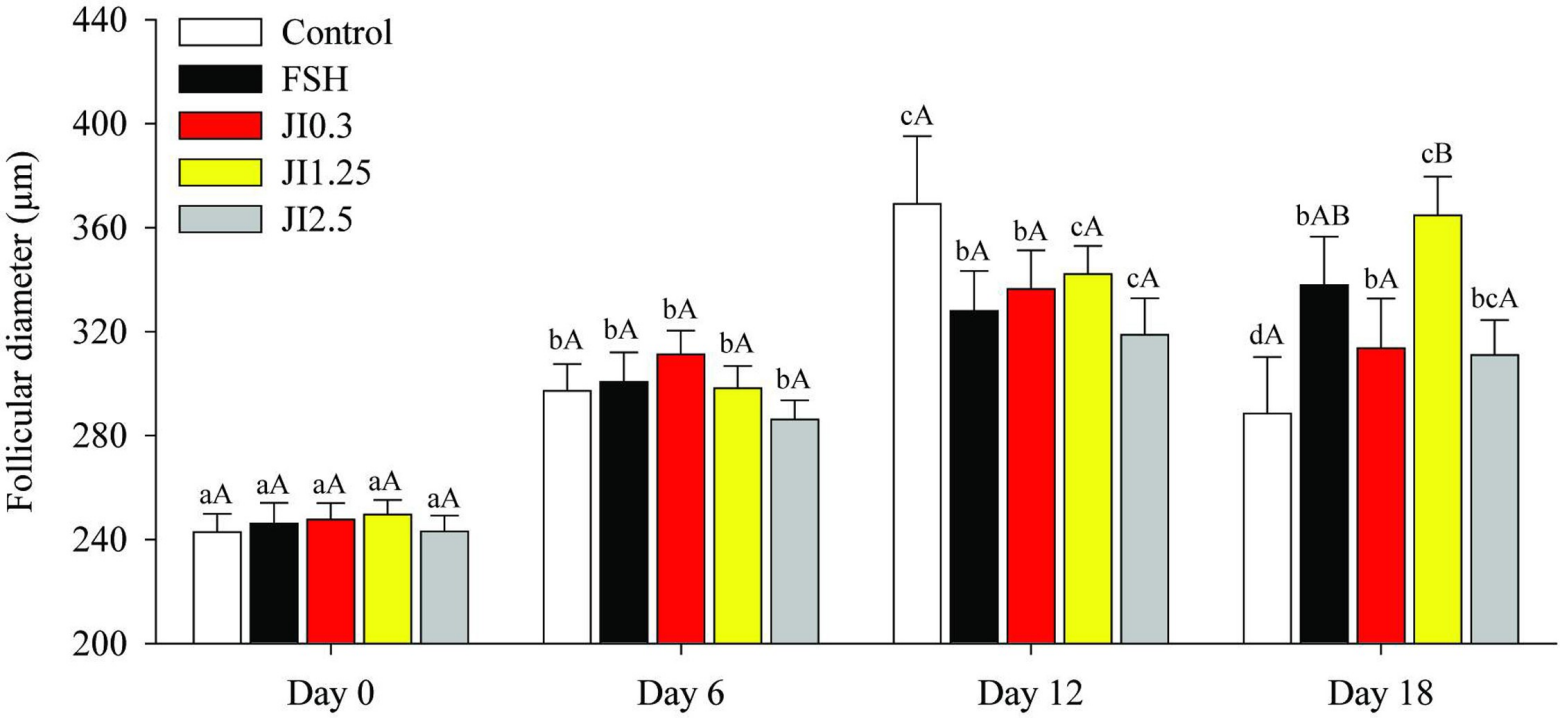
Follicles diameter at different time point (D0, D6, D12, D18) in MEM^+^ (Control), FSH or in *J*. *insularis* 0.3, 1.25 or 2.5 mg/mL (JI0.3, JI1.25 or JI2.5 respectively). D: day, MEM^+^: Minimal essential medium supplemented, FSH: recombinant bovine follicle stimulating hormone, JI: *J*. *insularis*. A total of 342 secondary follicles were distributed in the different treatments (n = 6 replicates). ^a,b,c,d^ Different letters denote significant differences among time for a given treatment group (P < 0.05). ^A,B^ Different letters denote significant differences among treatment groups within the same time point (P < 0.05).

Overall, follicular diameters progressively increased (P < 0.05) in all treatments until D12, except for FSH and J0.3 treatments in which diameter remained unchanged from D6 onwards. At D18, JI1.25 treatment led to a larger (P < 0.05) follicular diameter compared to control, JI0.3 and JI2.5 (Fig 3).

During the three intervals (D0—D6; D6—D12; D12—D18), growth rates reflected what we observed in Table 2 about the lack of differences between treatments until D12. The control, JI0.3 and JI1.25 treatments maintained growth rate until the second third of culture, although it dropped (P < 0.05) from the second (D6—D12) to the last third of culture (D12—D18). Comparing among treatments at D18, except JI0.3, all of them showed higher (P < 0.05) growth rate than control. Regarding the overall growth rate, none treatment differed from control. However, JI0.3 treatment showed a lower (P < 0.05) daily growth rate than all treatments, except the JI2.5 treatment.

**Table 2:** Mean (± SEM) daily growth of morphologically intact secondary follicles at different time point (D0, D6, D12, D18) in MEM^+^ (Control), FSH or in *J*. *insularis* 0.3, 1.25 or 2.5 mg/mL (JI0.3, JI1.25 or JI2.5 respectively).

Mean follicular growth (μm/day ± SEM) in different culture intervals				
Treatments	D0—D6	D6—D12	D12—D18	Overall
**Control**	10.3 ± 1.2^aA^	13.2 ± 2.4^aA^	2.5 ± 2.0^bA^	4.7 ± 0.6^AB^
**FSH**	9.7 ± 1.2^aA^	7.6 ± 1.7^abA^	4.8 ± 1.3^bB^	6.3 ± 0.8^A^
**JI0.3**	10.3 ± 0.9 ^aA^	7.1 ± 1.1^bA^	1.6 ± 1.8^cAB^	4.2 ± 0.6^B^
**JI1.25**	8.1 ± 0.7^aA^	7.2 ± 0.9^bA^	2.8 ± 0.9^cB^	5.6 ± 0.4^A^
**JI2.5**	7.1 ± 0.7^aA^	7.2 ± 1.1^aA^	4.9 ± 1.0^aB^	5.0 ± 0.3^AB^

^a,b,c^ Different letters denote significant differences among time for a given treatment group (P < 0.05).

^A,B^ Different letters denote significant differences among treatment groups within the same time point (P < 0.05).

JI: *J*. *insularis*. A total of 342 secondary follicles were distributed in the different treatments (n = 6 replicates).

Looking at the different follicular growth categories: non-growing, slow-growing and fast-growing follicles (Table 3), both treatments JI1.25 and JI2.5 presented, respectively, higher (P < 0.05) percentage of slow-growing follicles and lower (P < 0.05) percentage of fast-growing follicles than control.

**Table 3:** Percentage of secondary follicles within a growth category at different time point (D0, D6, D12, D18) in MEM^+^ (Control), FSH or in *J*. *insularis* 0.3, 1.25 or 2.5 mg/mL (JI0.3, JI1.25 or JI2.5 respectively).

	Growth category (%)*		
Treatments	Non growing	Slow-growing	Fast growing
**Control**	2.0 (1/50)^A^	58.0 (29/50)^A^	40.0 (20/50)^A^
**FSH**	3.9 (2/51)^A^	72.5 (37/51)^AB^	23.5 (12/51)^AB^
**JI0.3**	-	67.2 (4364)^AB^	32.8 (21/54)^A^
**JI1.25**	7.1 (4/56)^A^	78.6 (44/56)^B^	14.3 (8/56)^B^
**JI2.5**	2.8 (2/72)^A^	79.2 (57/72)^B^	18.0 (13/72)^B^

*In vitro*-cultured follicles were classified as: null, follicles that did not grow during culture; slow, follicles with a daily growth between 0.1 and 12.0 μm day –1 and fast, follicles with a daily growth between 12.1 and 46.7 μm day –1. ^A,B^ Different letters denote significant differences among treatment groups within the same time point (P < 0.05). JI: *J*. *insularis*. A total of 342 secondary follicles were distributed in the different treatments. We performed 6 replicates.

*Follicles were classified as: Non-growing, follicles that did not grow during the culture; Slow-growing, follicles with a daily growth rate between 0.1 and 12.0 μm/day; and fast-growing, follicles with a daily growth rate > 12.1 μm/day.

**Fig 4:**
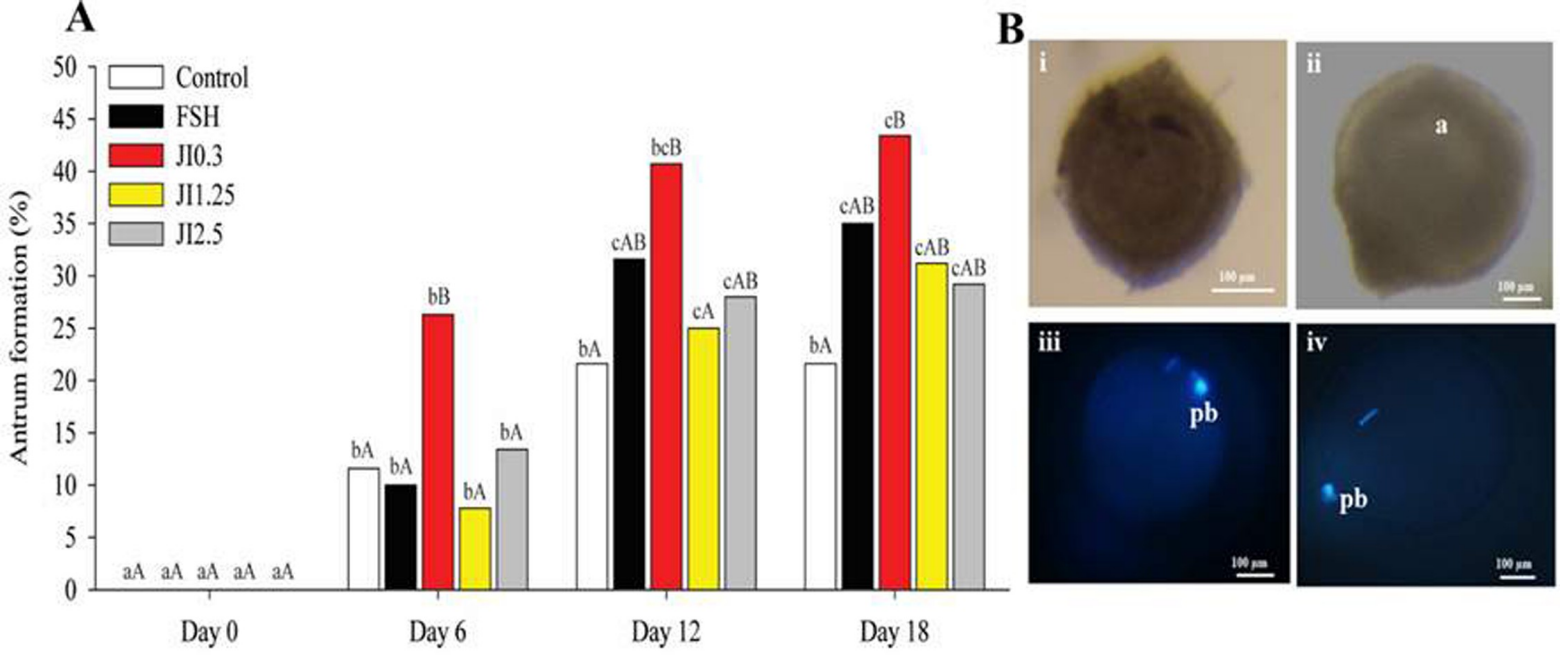
**Percentage of antrum formation (A) at different time point (D0, D6, D12, D18) in MEM**^**+**^ **(Control), FSH or in *J*. *insularis* 0.3, 1.25 or 2.5 mg/mL (JI0.3, JI1.25 or JI2.5 respectively). (B)**: **Normal sheep secondary follicle at D0 (i) or at D18 day in *J*. *insularis* 0.3 mg/mL (JI0.3) (ii). Note formation of antrum (a) on D18. Representative images of fluorescent metaphase II oocyte grown *in vitro* (iii) or *in vivo* (iv).** Note presence of the first polar body (cp). D: day, MEM^+^: Minimal essential medium supplemented, FSH: recombinant bovine follicle stimulating hormone, JI: *J*. *insularis*. A total of 342 secondary follicles were distributed in the different treatments (n = 6 replicates. ^a,b,c,d^ Different letters denote significant differences among time for a given treatment group (P < 0.05). ^A,B^ Different letters denote significant differences among treatment groups within the same time point (P < 0.05).

All treatments induced a progressive increase (P < 0.05) in the percentage of antral cavity formation compared to D0. Interestingly, at D6, JI0.3 treatment presented higher (P < 0.05) percentage of antral cavity formation than in the other treatments. In addition, JI0.3 was the only treatment that showed a higher (P < 0.05) percentage of antrum formation than control at any evaluated time point, although did not differ from the other treatments at the end of the culture or at D18 (Fig 4A).

Representative images of normal follicles, and metaphase II oocyte grown *in vitro* or *in vivo* are shown (Fig 4B).

### Influence of *J*. *insularis* extract on follicular extrusion during *in vitro* culture

**Fig 5:**
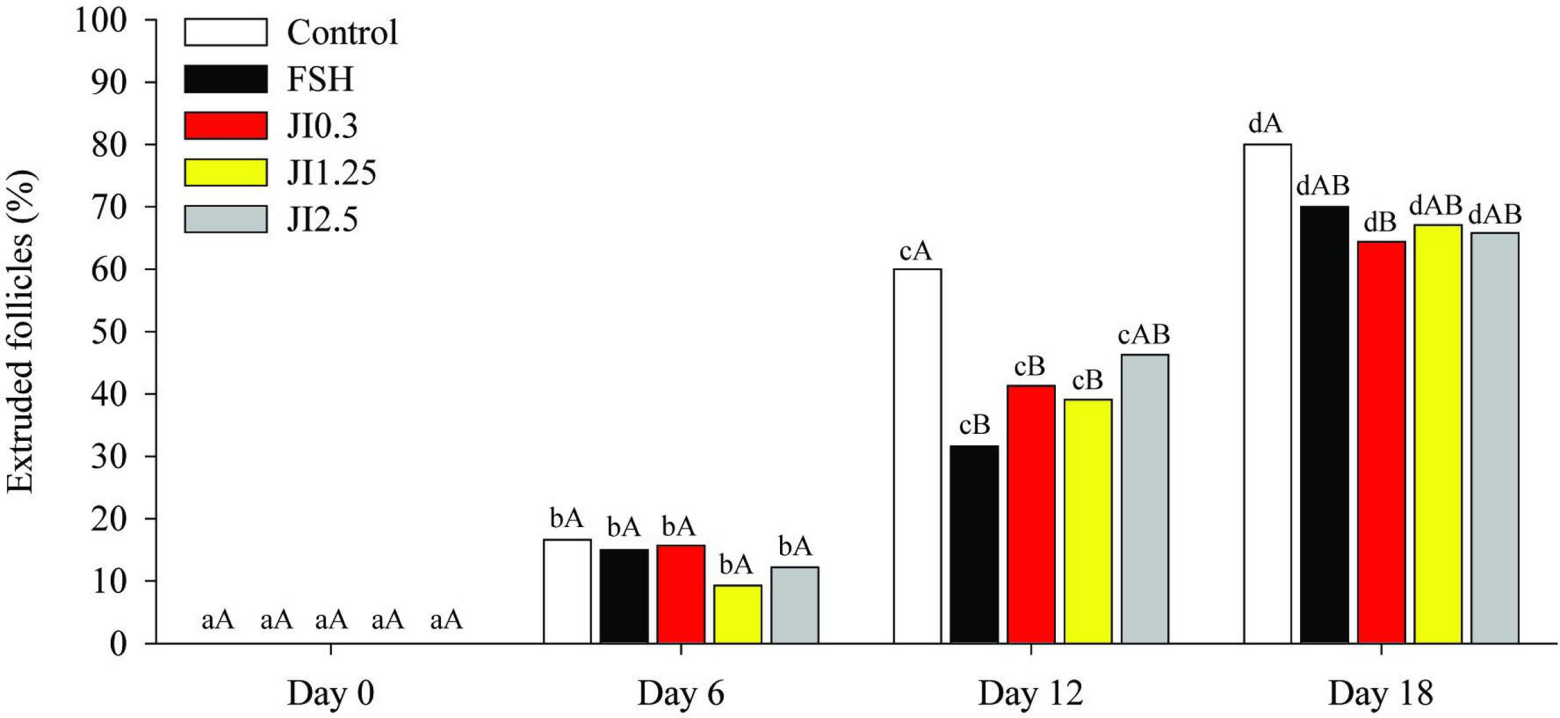
Percentage of extrusion at different time point (D0, D6, D12, D18) in MEM^+^ (Control), FSH or in *J*. *insularis* 0.3, 1.25 or 2.5 mg/mL (JI0.3, JI1.25 or JI2.5 respectively). D: day, MEM^+^: Minimal essential medium supplemented, FSH: recombinant bovine follicle stimulating hormone, JI: *J*. *insularis*. A total of 342 secondary follicles were distributed in the different treatments (n = 6 replicates). ^a,b,c,d^ Different letters denote significant differences among time for a given treatment group (P < 0.05). ^A,B^ Different letters denote significant differences among treatment groups within the same time point (P < 0.05).

The percentage of extruded follicles increased (P < 0.05) in all treatments from D0 to D18. Moreover, at D12, a higher percentage (P < 0.05) of extruded follicles was observed in the control compared to all treatments, except JI2.5. On the other hand, at D18, the percentage of extruded follicles in the control was higher (P < 0.05) only than JI0.3 (Fig 5).

#### Dynamic of follicular growth and extrusion of secondary follicles

Regardless the treatment, JI0.3 showed a fast-growing rate and a low percentage of extrusion compared to control. When comparing between follicle growth categories in different intervals of *in vitro* culture, the odds ratio (OR) analysis showed that this positive association was greater in fast-growing follicles compared to slow-growing follicles in the first two intervals of culture (D0—D6 and D6—D12) (P = 0.0001 and 0.0062, respectively) (Table 4).

**Table 4:** Association analyses between follicular growth categories and percentage of extrusion in different intervals of *in vitro* follicle culture.

Comparisons	Extrusion (%)		Odds ratio (CI 95%)		P-value	
	D0—D6	D6—D12	D0—D6	D6—D12	D0—D6	D6—D12
**Non-growing Slow-growing**	76.4(13/17) 62.9 (148/235)	45.8(11/24) 46.8(66/141)	1.9 (0.6–6.0)	1.0 (0.4–2.4)	0.3915	0.8944
**Non-growing Fast-growing**	76.4 (13/17) 85.1 (80/94)	45.8 (11/24) 69.6 (39/56)	1.7 (0.5–6.1)	2.7 (1.0–7.2)	0.5951	0.0778
**Slow-growing Fast-growing**	62.9 (148/235) 85.1 (80/94)	46.8 (66/141) 69.6 (39/56)	3.3 (1.7–6.2)	2.6 (1.3–5.0)	0.0001	0.0062

*In vitro*-cultured follicles were classified as: null, follicles that did not grow during culture; slow, follicles with a daily growth between 0.1 and 12.0 μm day ^–^1 and fast, follicles with a daily growth between 12.1 and 46.7 μm day ^–^1.

### Influence of *J*. *insularis* extract on levels of ROS during the *in vitro* culture of follicles

Differences among treatments were not found at D6. On the other hand, at D18 all treatments presented higher (P < 0.05) ROS concentration than the control. In addition, JI2.5 presented a higher (P < 0.05) levels of ROS than FSH, although did not differ from JI0.3 or JI1.25 (Table 5).

**Table 5:** Level of ROS measured in the culture medium from secondary follicles before (D0) and during 18 days of *in vitro* culture (D6, D12, D18) in MEM^+^ (Control), FSH or in JI0.3, JI1.25 or JI2.5.

Treatments	Day 6	Day 18
**Control**	14.2 ± 2.5^A^	11.1 ± 0.2^A^
**FSH**	14.6 ± 2.5^A^	13.5 ± 0.6^B^
**JI0.3**	14.9 ± 0.8^A^	14.5 ± 0.7^BC^
**JI1.25**	14.5 ± 0.9^A^	15.1 ± 1.7^BC^
**JI2.5**	16.1 ± 2.0^A^	17.3 ± 1.9^C^

^A,B,C^ Within a column, values without a common superscript differed (P < 0.05).

### Influence of *J*. *insularis* extract on gene expression in follicular walls during *in vitro* culture

**Fig 6:**
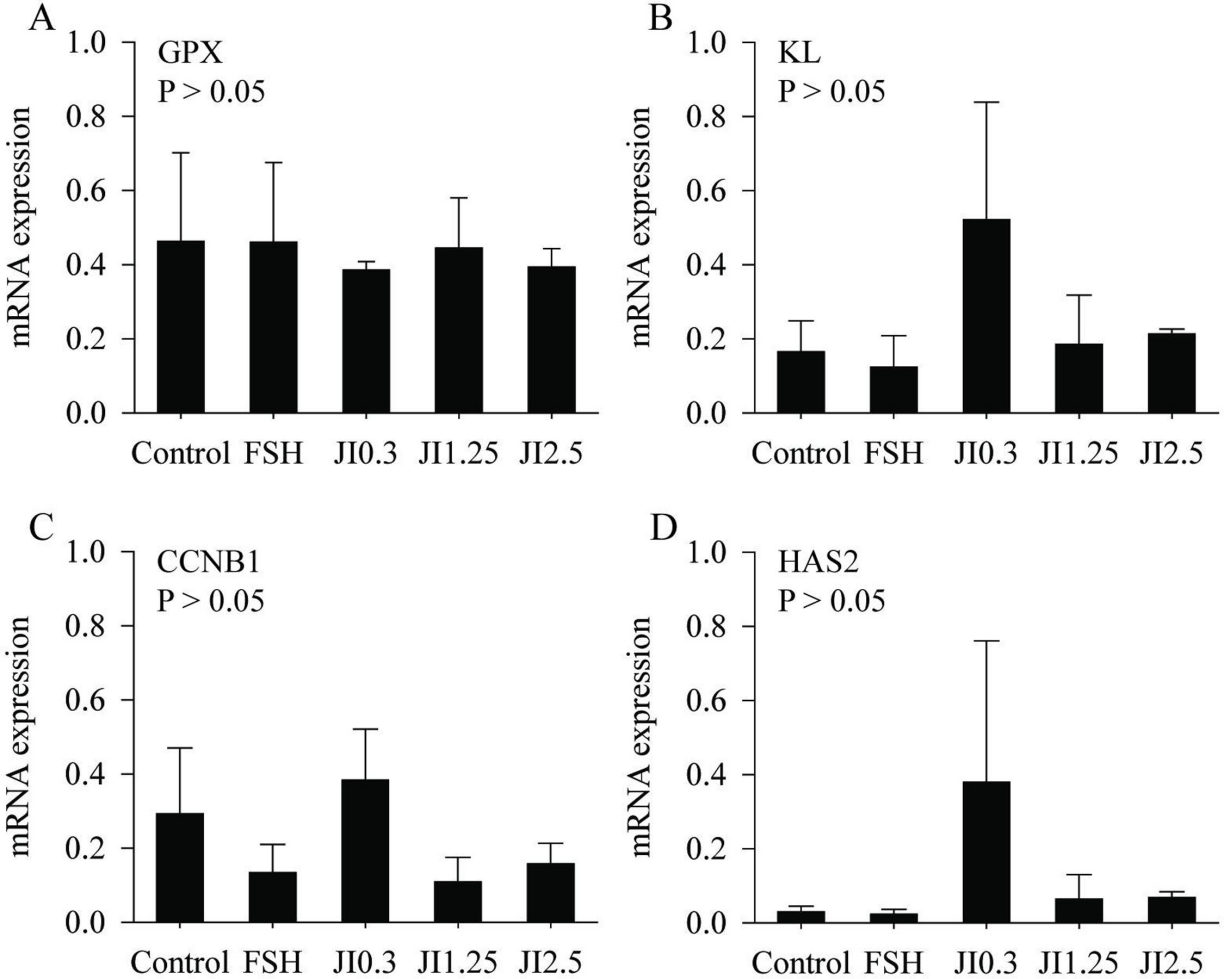
**Relative mean (standard error of the mean) expression of mRNA of GPX (A), KL (B), CCNB1 (C), and HAS2 (D) before (D0) and during 18 days of *in vitro* culture (D6, D12, D18) in MEM**^**+**^ **(Control), FSH or in JI0.3, JI1.25 or JI2.5**. ^A,B^ Different letters denote significant differences among treatments (P < 0.05).

The relative expression of GPX, KL, CCNB1, and HAS2 was measured in follicular walls. No differences were observed between control and all treatments in mRNA expression for GPX, KL, CCNB1 and HAS2 (Fig 6A, 6B, 6C and 6D respectively).

### Influence of *J*. *insularis* extract on structure and function of oocytes resulting from follicle cultured *in vitro*

In the first experiment, the percentage of fully grown oocytes (≥110 μm), oocyte diameter, viability rate and meiotic stages after IVM are shown in Table 6. None treatment differed from control in any evaluated end point. However, the viability rate was significantly higher in JI0.3 compared to FSH treatment.

**Table 6:** Percentage of fully grown oocytes, oocyte diameter, viability rate, percentage of meiotic resumption, and meiotic stages of oocytes matured in TCM 199 (Control), FSH or in *J*. *insularis* 0.3, 1.25 or 2.5 mg/mL (JI0.3, JI1.25 or JI2.5 respectively).

Treatments	(%)Fully grown oocytes (≥110 μm/n)	Oocyte diameter (Mean±SEM)	(%)Viability (viable/n)	(%) Meiotic resumption (n/viable)	(%)GV (n/viable)	(%) GVBD (n/viable)	(%)MI (n/viable)	(%) MII (n/viable)
**Control (n = 43)**	30.2 (13/43)^A^	106.8 ± 2.9^A^	76.7 (33/43)^AB^	42.2 (14/33)^A^	54.6 (18/33)^A^	18.2 (6/33)^A^	15.2 (5/33)^A^	9.1 (3/33)^A^
**FSH (n = 45)**	37.8 (17/45)^A^	106.8 ± 2.5^A^	64.4 (29/45)^A^	41.4 (12/29)^A^	58.6 (17/29)^A^	17.2 (5/29)^A^	13.8 (4/29)^A^	10.3 (3/29)^A^
**JI0.3 (n = 45)**	35.5 (16/45)^A^	105.0 ± 3.5^A^	84.4 (38/45)^B^	36.8 (14/38)^A^	63.2 (24/38)^A^	15.8 (6/38)^A^	10.5 (4/38)^A^	10.5 (4/38)^A^
**JI1.25 (n = 44)**	36.3 (16/44)^A^	108.1 ± 1.9^A^	68.2 (30/44)^AB^	40.0 (12/30)^A^	63.3 (19/30)^A^	20.0 (6/30)^A^	10.0 (3/30)^A^	10.0 (3/30)^A^
**JI2.5 (n = 40)**	30.0 (12/40)^A^	105.1 ± 3.8^A^	75.0 (30/40)^AB^	46.7 (14/30)^A^	56.7 (17/30)^A^	26.7 (8/30)^A^	6.7 (2/30)^A^	13.3 (4/30)^A^

^A,B^ Different letters denote significant differences among treatment groups within the same time point (P < 0.05).

TCM: Tissue culture media 199, FSH: recombinant bovine follicle stimulating hormone, JI: *J*. *insularis*. A total of 217 complexes oocyte cumulus cells from secondary follicles grown *in vitro* were matured (n = 6 replicates).

After IVM of oocytes originate from follicles grown in vivo (Control experiment), the percentage of oocyte viability and meiotic resumption (n/total) was significantly higher (P < 0.05) in JI1.25 compared to JI2.5. Regarding to the percentage of metaphase II oocytes, FSH showed a similar percentage than JI0.3. Moreover, only JI0.3 showed a significant higher (P < 0.05) percentage than control (Table 7).

**Table 7:** Percentage of oocyte viability, meiotic resumption, and meiotic stages of oocytes recovery from antral follicles matured in TCM 199 (Control), FSH or in *J*. *insularis* 0.3, 1.25 or 2.5 mg/mL (JI0.3, JI1.25 or JI2.5 respectively).

Treatments	(%) viability (viable/n)	(%) Meiotic resumption (n/total)	(%) Meiotic resumption (n/viable)	(%) VG (n/viable)	(%) GVBD (n/viable)	(%) MI (n/viable)	(%) MII (n/viable)
**Control (n = 65)**	58.5 (38/65)^AB^	52.3 (34/65)^AB^	89.5 (34/38)^A^	10.5 (4/38)^A^	23.7 (9/38)^A^	44.7 (17/38)^A^	21.1 (8/38)^A^
**FSH (n = 59)**	59.3 (35/59)^AB^	54.2 (32/59)^AB^	91.4 (32/35)^A^	8.6 (3/35)^A^	22.9 (8/35)^A^	40.0 (14/35)^A^	28.6 (10/35)^AB^
**JI0.3 (67)**	58.2 (39/67)^AB^	56.7 (38/67)^AB^	97.4 (38/39)^A^	2.6 (1/39)^A^	25.6 (10/39)^A^	28.2 (11/39)^A^	43.6 (17/39)^B^
**JI1.25 (66)**	51.5 (34/66)^A^	47.0 (31/66)^A^	91.2 (31/34)^A^	8.8 (3/34)^A^	29.4 (10/34)^A^	26.5(9/34)^A^	35.3 (12/34)^AB^
**JI2.5 (71)**	67.6 (48/71)^B^	63.4 (45/71)^B^	93.8 (45/48)^A^	6.2 (3/48)^A^	25.0 (12/48)^A^	43.8 (21/48)^A^	25.0 (12/48)^AB^

^A,B^ Different letters denote significant differences among treatment groups within the same time point (P < 0.05).

TCM: Tissue culture media 199, FSH: recombinant bovine follicle stimulating hormone, JI: *J*. *insularis*. A total of 328 complexes oocyte cumulus cells from antral follicles grown *in vivo* were mature (n = 3 replicates)

## Discussion

To the best of our knowledge, this study constitutes the first report demonstrating the beneficial effects of the *J*. *insularis* extract on the *in vitro* culture and maturation of ovine isolated follicles. While FSH has been widely used to maintain follicle survival [41], promote the antrum formation [42] and stimulate follicular growth and oocyte maturation [11, 36], similar effects were obtained with 0.3 mg/mL of *J*. *insularis*. Addition of this natural substance in the culture or maturation medium represents an efficient alternative to FSH.

The phytochemical screening by TLC of the aqueous extract of *J*. *insularis* revealed alkaloids and flavonoids. In addition, a sample (1.0 g) of the aqueous extract was fractionated on sephadex LH-20 eluted with MeOH 8:2 to furnish four fractions which were analyzed by ^1^H NMR and LC-MS. Inspection of these techniques allowed to identify the alkaloid trigonelline [43] and phenol compounds as eucomic acid [44] and kaempferol glycosides derivatives [45] in agreement with TLC. As expected, sugars as sucrose, and the α- and β-glucose stereoisomers [46] were also identified. Although there are no studies on the isolation of the chemical constituents of *J*. *insularis*, a literature survey on *Justicia* species revealed the presence of those classes of compounds [29], corroborating with our findings (S2 Fig). In addition, according to the literature, plants of the genus *Justicia* are known as producers of bioactive compounds as alkaloids and flavonoids [47].

We should take into account that the seasonality of climatic elements such as temperature, relative humidity and solar radiation can alter the physiological behavior of plants and, consequently, their growth and development, as well as the chemical and biological composition of the soil [48]. Thus, for all the secondary metabolites identified from *J*. *insularis*, the variation of climatic elements can affect their concentrations in the plant [49]. Therefore, the environments in which the plant develops exert a direct influence on the chemical composition of the extracts.

In our study, percentages of morphologically intact follicles decreased at the end of the culture period regardless of the treatment. This expected result is common during *in vitro* culture of secondary follicles [12, 19]. Such effect could be due to the action of toxic metabolites and ROS. In excess, these metabolites can oxidize important molecules that induce the release of nucleases, proteases, and lipases from mitochondria [12], resulting in the disruption of the basal membrane [50]. This was observed in previous published articles using natural substances with the same culture period in ovine [22, 24] and goat species [19, 23]. Interestingly, *J*. *insularis* at 0.3 mg/mL showed a higher percentage of intact follicles than the control. This could be due to the action of the flavonoids 3’-Metoxy-kaempferol-arabinosyl-rhamnoside, and Kaempferol-arabinosyl-rhamnoside which are phenolic compounds identified in the extract and have the capacity to neutralize damage caused by oxidation. Choi, [51] has demonstrated that pretreatment with kaempferol prior to antimycin A exposure significantly reduced cell damage by preventing mitochondrial membrane potential dissipation and ROS production. Other authors showed that cellular reactive oxygen species levels distinctly diminished by trigonelline treatment of HT-29/Caco-2 cells [52].

Percentages of extrusion increased significantly during culture period in all treatments. Moreover, the JI0.3 showed lower percentage of extruded follicles compared to control. This may be due to the increased in the follicular growth. Indeed, analyses of the chances of extrusion in non, slow or fast-growing follicles within the first (D0—D6) and second (D6—D12) third of the culture *in vitro* showed that in the first third (D0—D6), fast-growing follicles were 3.3 times more likely to extrude than those with slow growth. Furthermore, in the second third (D6—D12), the same behavior occurred (fast-growing follicles were 2.6 times more likely to extrude than slow-growing follicles).

During the culture period, all treatments increased follicle diameter. Interestingly, FSH and JI0.3 treatments showed similar follicle diameter. FSH has a well-established role in modulating granulosa cell proliferation and antrum formation which contributed to an increase in follicular diameter [53]. Erickson et al. [54] reported that FSH interacts with several growth factors such as KL to induce follicular growth. Luz et al. [21] showed that culture medium of ovine secondary follicles supplemented with KL resulted in an increased rate of follicular diameter and antrum formation. The JI 0.3 response similar to FSH could be due to the action of the trigonelline which is a phytohormone that induced the proliferation of neuronal cells [55]. How trigonelline acts on the receptor of granulosa cells awaits further investigations.

All the treatments increased the antrum formation. Such effect could be due to the secondary metabolites of the plant. The TLC, ^1^H NMR and LC-MS analysis revealed the presence of alkaloid, flavonoids and glycosylated terpenoids. These metabolites are responsible for enhancing ovarian follicle growth and increasing in the number of corpus luteum in rats [33], and promoted the *in vitro* activation and survival of ovine preantral follicles enclosed in ovarian tissue [32]. Interestingly, at D6, JI0.3 showed a higher percentage of antrum formation. This finding is important because the ability to form an antrum is considered as a good marker of follicular functionality [56], as the mechanisms by which small cavities of fluid develop inside the follicle to form the antral cavity are related to the secretion of osmotically active molecules into small spaces between the granulosa cells [57]. Recently, a study using *Amburana cearensis* showed that its metabolite protocatechuic acid improves the antrum formation after 18 days of ovine preantral follicle culture *in vitro* [22].

In the current study, JI0.3 caused a fast-growing of secondary follicles, however, this treatment resulted in low percentage of extrusion compared to control. This means that there was an adequate response of the oocytes without membrane damages. Similar results were reported in sheep secondary follicles, grown for 6 days with the same culture medium [58]. This may be due to the interaction between flavonoids present in the plant extract and components of the culture medium. It is known that the flavonoids protect human vascular endothelial cells against oxidative damages [59] and have antimicrobial and antioxidant properties [60]. These flavonoids interact with membrane proteins, making them stable. Therefore, we believe that this increasing hardness can give follicular membrane firmness and prevent its rupture, consequently avoiding oocyte extrusion. On the other hand, other studies using androstenedione and FSH in the culture medium of isolated caprine secondary follicles found that higher percentage of fast-growing follicles was detrimental for efficiency of caprine secondary follicles cultured *in vitro* [38].

Recent studies have shown that *J*. *insularis* maintains the ROS level [32], however, in the current study at D18, all treatments presented higher ROS concentration than control. We believe that these results may be attributed to an excess of antioxidants in the culture medium. It is known that the balance between ROS and antioxidants within the oocytes is critical to cell functions, such as chromosome segregation [61], mitochondria activity [62], ATP level maintenance and DNA methylation [63]. Therefore, ROS may affect follicles and oocyte growth during the *in vitro* culture. Indeed, our basic culture medium is rich in supplements among which selenium (5.5 μg/mL) and transferrin (50 ng/ mL). These supplements in addition to *J*. *insularis* increases the ROS level. In this study, the increase of the ROS level was not detrimental for the follicles because no significant difference was observed on the GPx mRNA expression.

Several genes have been identified in follicular walls as biomarkers to understand the dynamics of follicular development. Among them GPx related to oxidative stress [64], KL indicative of follicular growth [65]; cyclin B1 and HAS2 indicative of progression of oocyte maturation [66]. We observed that after the IVM of COCs from secondary follicles, the follicular walls (granulosa and theca cells) expressed similar transcript levels. The gene expression similarity between J0.3 and FSH may support the use of this natural substance for the *in vitro* culture of ovine isolated secondary follicles.

Despite no effect was observed on the meiotic resumption of oocytes from *in vitro* growth secondary follicles, addition of JI0.3 on the maturation medium of oocyte from antral follicles showed similar percentage than FSH. In addition, this percentage was higher than the control. We believe that the presence of *J*. *Insularis* in the maturation medium could have improve the synthesis of the mitosis-promoting factor (MPF). In fact, during the meiosis resumption, the MPF, a protein complex composed of subunits cyclin B1 and p34cdc2 [67] is activated and regulate the germinal vesicle breakdown (GVBD) [68]. MPF activity is regulated by the CDK1 produced by the cells. The activity of MPF was described in many mammalian oocytes: it appears just before GVBD and increases until metaphase I stage, then its activity decreases in anaphase-telophase and increases again, reaching its maximum level in metaphase II in goat [69], sheep [70] and cow [71]. The oocyte (recovery from antral follicles) viability rate of was higher in JI1.25 compared to JI2.5. This could be due to an excess of toxic metabolites and reactive oxygen species present of the maturation medium. In fact, phytochemical analysis of the extract showed that it is constitute of secondary metabolites (flavonoids 3’-Metoxy-kaempferol-arabinosyl-rhamnoside, and Kaempferol-arabinosyl-rhamnoside, trigonelline among others) which have antioxidant activity as revealed by previous study (Bakuradze et al., 2010; Choi et al., 2011). In high concentration, those metabolite could acts as pro-oxidant and therefore increase the level of reactive oxygen species (free radical, hydrogen peroxide, hydroxyl ion…). As reported by Costa et al., 2011, although a critical amount of reactive oxygen species is essential for the physiological activities of follicles, excessive amount of them generates a contrary effect.

In summary, the present study showed for the first time that 0.3 mg/mL of *J*. *insularis* showed similar effect than FSH when added in the culture or maturation media of ovine isolated secondary follicles. It seems that when FSH is not available, *Justicia iusularis* could be used with the same benefits. Further studies are needed to isolate the metabolites present in *J*. *insularis* and assess the developmental competence of the oocytes after IVF.

## Supporting information

S1 Fig Identification of secondary metabolites by thin layer chromatography (TLC). The developers used are specific to each class of metabolite as described: (A) specific Drangendorffi reagent to identify alkaloids; (B) α-Naphthol acid solution for glycosylated compounds; (C) acid solution of vanillin; (D) solution of ethanol / sulfuric acid used as universal developers and (E) acid solution of cerium sulfate for flavonoids and terpenoids.
(PDF)

S2 Fig Metabolites of *J*. *insularis* identified by proton nuclear magnetic resonance (^1^H NMR) and liquid chromatography-mass spectrometry (LC-MS). (PDF)
