# Supplementary figures and images for: Supplementation of in vitro culture medium with FSH to grow follicles and mature oocytes can be replaced by extracts of Justicia insularis
Source: PLoS One. 2018 Dec 7;13(12):e0208760. doi: 10.1371/journal.pone.0208760 (PMC6286020; doi:10.1371/journal.pone.0208760)

**Fig. Identification of secondary metabolites by thin layer chromatography (TLC).**

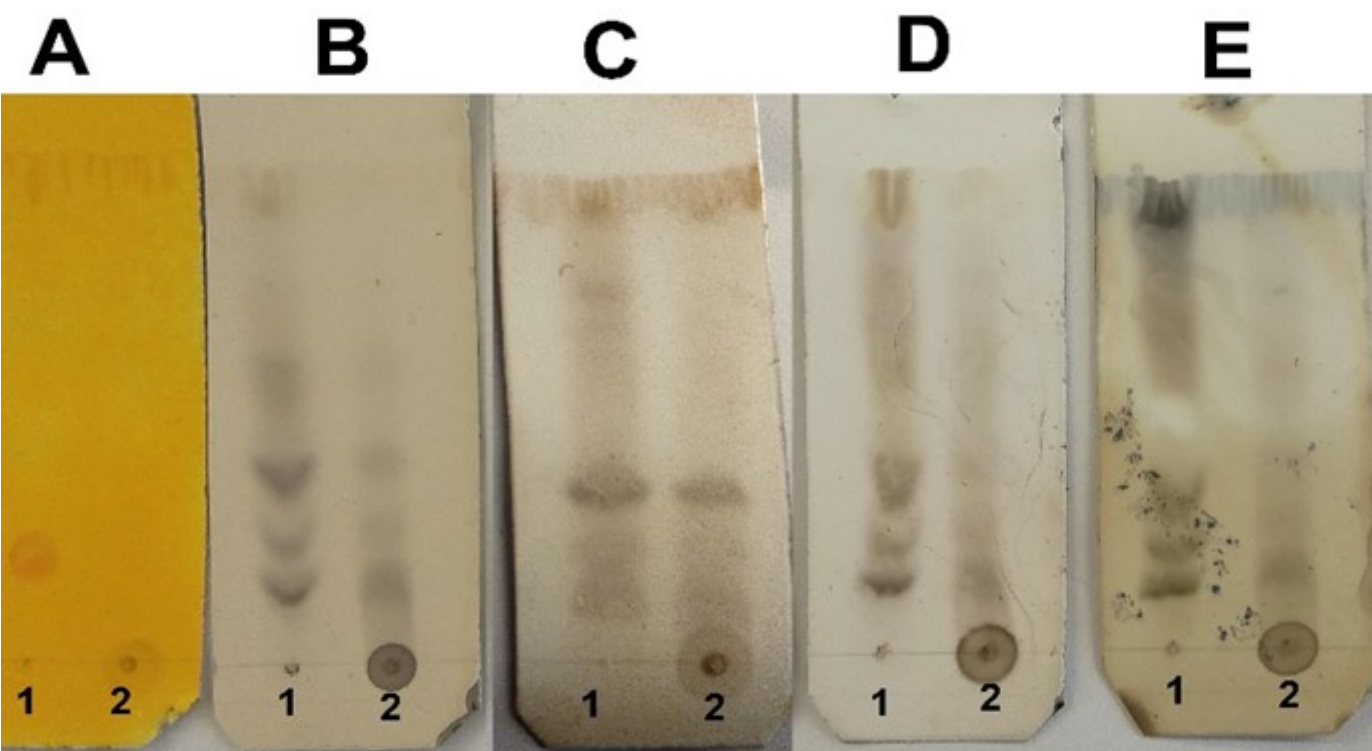

Supplement: S1 Fig — The developers used are specific to each class of metabolite as described: (A) specific Drangendorffi reagent to identify alkaloids; (B) α-Naphthol acid solution for glycosylated compounds; (C) acid solution of vanillin; (D) solution of ethanol / sulfuric acid used as universal developers and (E) acid solution of cerium sulfate for flavonoids and terpenoids. (PDF) [file pone.0208760.s001.pdf]
