# Supplementary material for: Supplementation of in vitro culture medium with FSH to grow follicles and mature oocytes can be replaced by extracts of Justicia insularis
Source: PLoS One. 2018 Dec 7;13(12):e0208760. doi: 10.1371/journal.pone.0208760 (PMC6286020; doi:10.1371/journal.pone.0208760)

S2 Fig. Metabolites of *J. insularis* identified by proton nuclear magnetic resonance (^{1}H NMR) and liquid chromatography-mass spectrometry (LC-MS).

^{1}H NMR (300 MHz in D_2O) fraction **JI-1**

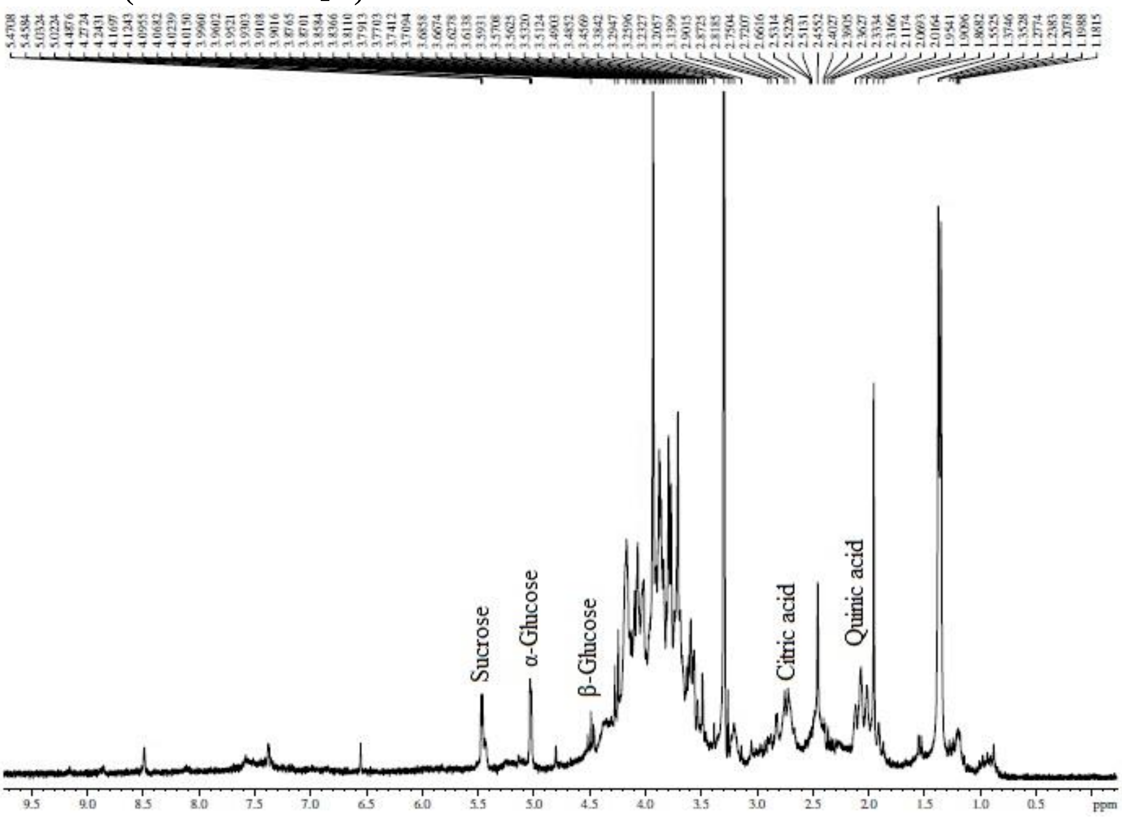

^{1}H NMR (300 MHz in D_2O) fraction **JI-2**

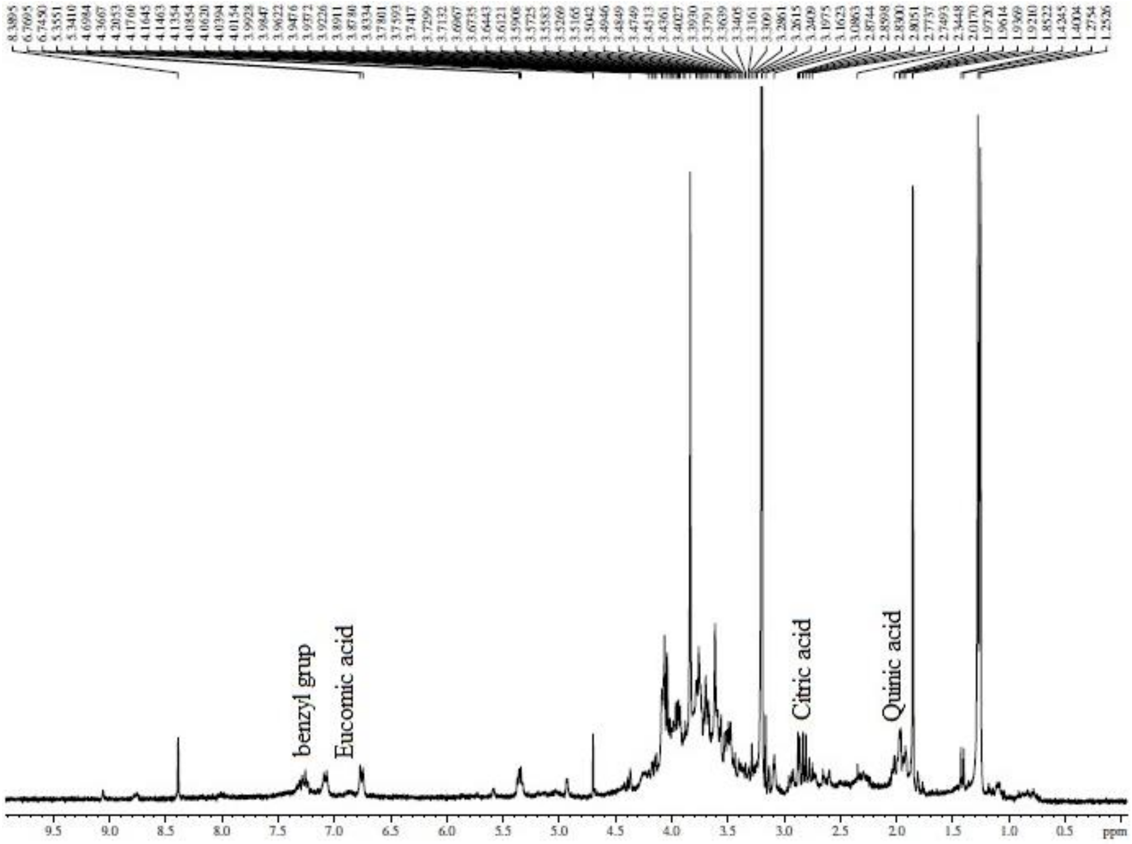

^{1}H NMR (300 MHz in MeOD) fraction **JI-3**

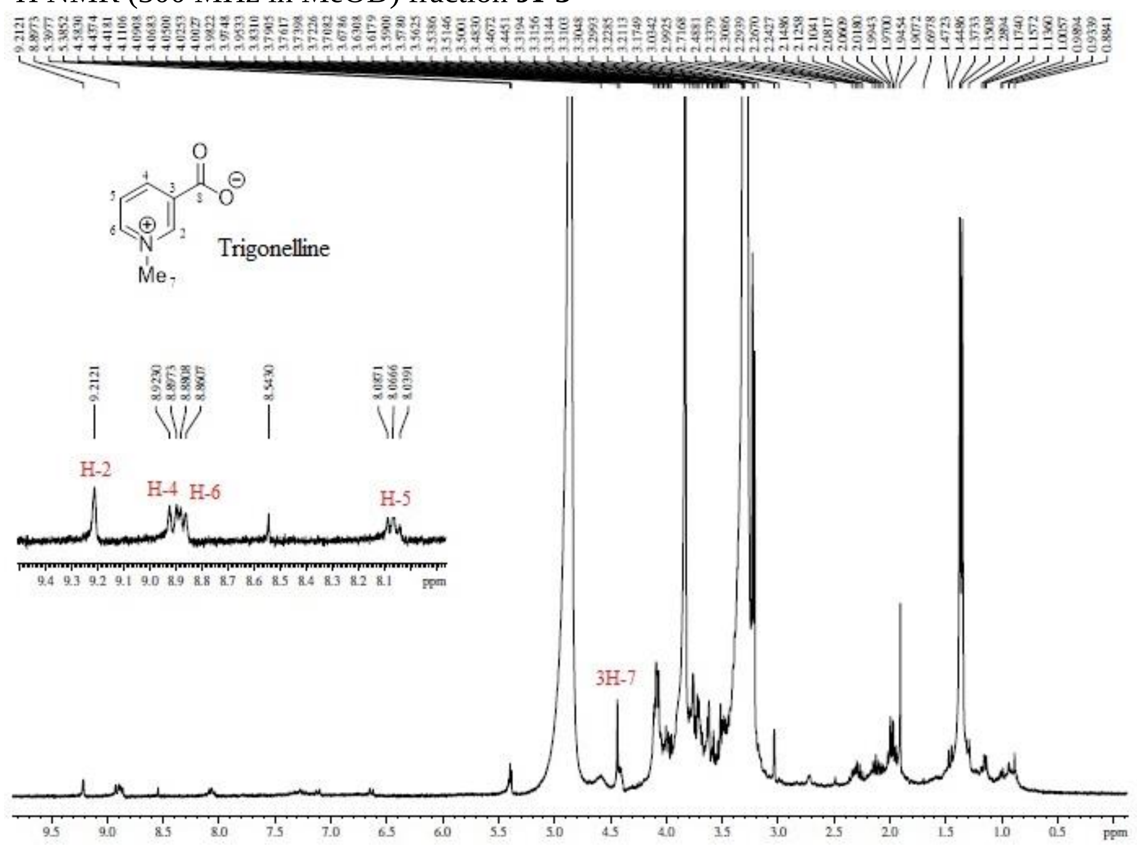

^{1}H NMR (300 MHz in MeOD) fraction **JI-4**

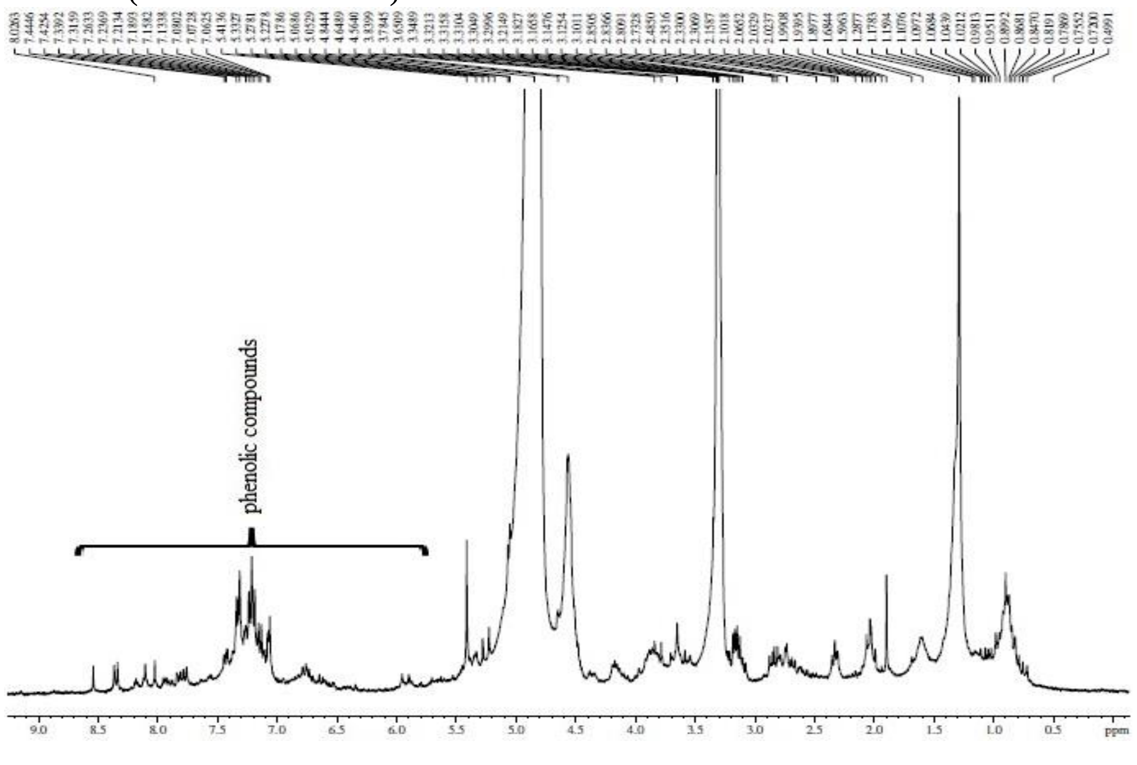

3’-Metoxy-kaempferol-arabinosyl-rhamnoside

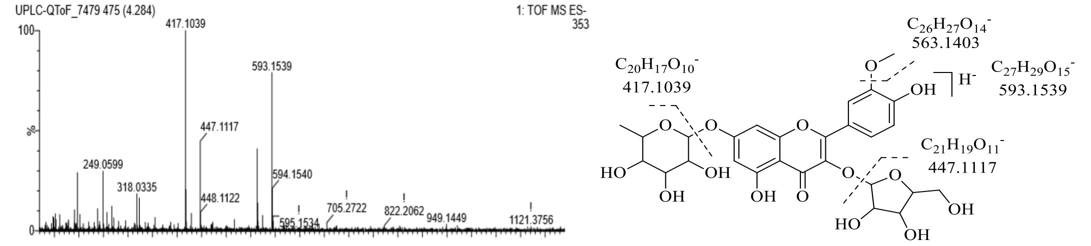

Kaempferol-arabinosyl-rhamnoside

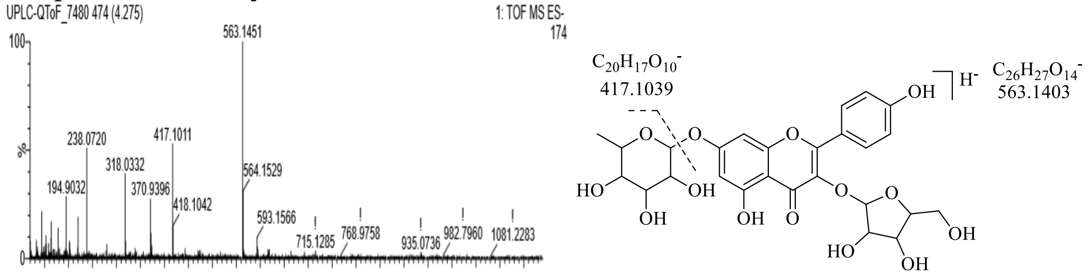

Supplement: S2 Fig — (PDF) [file pone.0208760.s002.pdf]
